# Homeopathic treatment of elderly patients - a prospective observational study with follow-up over a two year period

**DOI:** 10.1186/1471-2318-10-10

**Published:** 2010-02-22

**Authors:** Michael Teut, Rainer Lüdtke, Katharina Schnabel, Stefan N Willich, Claudia M Witt

**Affiliations:** 1 Institute for Social Medicine, Epidemiology and Health Economics, Charité University Medical Center, Luisenstr. 57, D-10017 Berlin, Germany; 2 Karl und Veronica Carstens Foundation, Am Deimelsberg 36, D-45276 Essen, Germany

## Abstract

**Background:**

Very little is known about the range of diagnoses, course of treatment and long-term outcome in elderly patients who choose to receive homeopathic medical treatment. We investigated homeopathic practice in an industrialised country under everyday conditions.

The aim of the study was to determine the spectrum of diagnoses and treatments, as well as to describe the course of illness over time among older patients who chose to receive homeopathic treatment.

**Methods:**

In this subgroup analysis of a prospective, multicentre cohort study totally including 3981 patients treated by homeopathic physicians in primary care practices in Germany and Switzerland, data was analysed from all patients > 70 years consulting the physician for the first time. The main outcome measures were: assessment by patient of the severity of complaints (numeric rating scales) and quality of life (SF-36) and by the physician of the severity of diagnoses (numeric rating scales) at baseline, and after 3, 12, and 24 months.

**Results:**

A total of 83 patients were included in the subgroup analysis (41% men, mean age 73.2 ± (SD) 3.1 years; 59% women, 74.3 ± 3.8 years).

98.6 percent of all diagnoses were chronic with an average duration of 11.5 ± 11.5 years. 82 percent of the patients were taking medication at baseline.

The most frequent diagnoses were hypertension (20.5%, 11.1 ± 7.5 years) and sleep disturbances (15.7%, 22.1 ± 25.8 years).

The severity of complaints decreased significantly between baseline and 24 months in both patients (from 6.3 (95%CI: 5.7-6.8) to 4.6 (4.0-5.1), p < 0.001) and physicians' assessments (from 6.6 (6.0-7.1) to 3.7 (3.2-4.3), p < 0.001); quality of life (SF 36) and the number of medicines taken did not significantly change.

**Conclusion:**

The severity of disease showed marked and sustained improvements under homeopathic treatment, but this did not lead to an improvement of quality of life. Our findings might indicate that homeopathic medical therapy may play a beneficial role in the long-term care of older adults with chronic diseases and studies on comparative effectiveness are needed to evaluate this hypothesis.

## Background

Homeopathy is one of the most frequently used but controversial forms of complementary and alternative medicine (CAM). It is based on the ancient 'principle of similars'. Highly diluted preparations of substances that cause symptoms in healthy individuals are used to stimulate healing reactions in patients who display similar symptoms when ill [1]. In classical homeopathy a single homeopathic remedy is selected, based on a patient's total spectrum of symptoms [2]. The proportion of patients obtaining homeopathic care has quadrupled in the last seven years according to a US survey [3]. For Germany a recent survey demonstrated that approximately 10% of men and 20% of women in the general population used homeopathic medicines during the previous year [4]. To date, few studies have evaluated the use of complementary therapies in geriatric patients [5]. In Germany, 73% of adults over 60 years of age use naturopathic drugs [6,7]. There is little information about the safety and efficacy of these interventions, especially if they are combined with conventional therapies [8].

This project was designed with the goal of systematically collecting data in this area of homeopathic health care for the first time in Germany.

The aim of the present study was to determine the spectrum of diagnoses and treatments, as well as to describe the course of illness over time among older patients who chose to receive homeopathic treatment. To collect data on the use and effects of homoeopathy under conditions of usual care, we investigated 3981 patients in a prospective observational study [9-11]. This paper presents the results of our evaluation, focusing on a subgroup of 83 adults over 70 years of age, who consulted a homeopathic physician.

## Methods

### Study design

In a prospective, multicentre cohort study involving 38 primary care practices with additional specialisation in homeopathy in Germany and Switzerland, in this subgroup analysis data was analysed from all patients being 70 years or older consulting the physician for the first time. Patients were included consecutively at their first consultation with a participating physician and were followed up for a total of 24 months. In order to provide as representative a picture of homeopathic health care as possible, patients were included in the study regardless of their diagnosis. Sixty-eight percent of all approached patients agreed to participate in the study. For description of the selection process see [10].

In order to participate in the study, physicians were required to have certified training in classical homeopathy and at least three years practical experience, they all followed the principles of classical homeopathy. All homeopathic physicians worked in their own doctor's offices, hospital services were not included. A total of 187 physicians belonging to four different working groups were contacted either by post or telephone and informed about the study. Of these, 103 physicians chose to participate.

All study participants provided written, informed consent, and the study protocol was approved by the appropriate ethics review boards [10].

### Treatments

To reflect usual care, all homeopathic physicians were completely free to choose a treatment. This usually included the prescription of homeopathic medicines according to homeopathic principles, but could also include the prescription, change or withdrawal of a conventional medicine, referrals to specialists, or admission to a hospital.

### Outcome measures

All questionnaires were designed to document sociodemographic data, as well as information on prior medical history, patient symptoms and complaints, quality of life, and the use of any treatment other than homeopathy. At baseline, patients recorded the complaints that led them to consider homeopathic treatment. Independently of their physicians, patients rated the severity of their complaints as they experienced them on a numeric rating scale (NRS, 0 = no complaints, 10 = maximum severity of complaints the patient could imagine for this disease) [12]. All complaints listed by patients in their baseline questionnaire were transferred to their follow-up questionnaires by the study office personnel, which ensured that each baseline complaint was assessed at each subsequent follow-up. General health-related quality of life was assessed using the MOS SF-36 questionnaire [13]. The results of the SF-36 are presented in normalised scores, the results being scaled in such a way that the normal German population, in the age group considered, has a mean score of 50 and a standard deviation of 10. (As quality of life is considerably lower in this age group than in the whole population this normalisation should not be confused with a normalisation of the whole German population).

The first questionnaire was distributed to the patients by the homeopathic physician treating the patient and completed prior to the start of therapy (baseline). The ensure this physicians and their nurses were trained in the in the process and a monitor visited the practice to check the process during the study. Patients sent their completed questionnaires to the study office in sealed envelopes. Follow-up questionnaires were sent to all patients by the study office at 3, 12, and 24 months.

For physicians, we developed a standardised questionnaire that allowed for continuous documentation during the treatment/follow-up period (24 months), as well as standardised points of assessment at 0, 3, 12 and 24 months. At each of these time points the physician saw the patient and estimated the severity of a maximum of four diagnoses from their perspective after they did the case taking with the patient using a numeric rating scale (NRS, 0 = no complaints, 10 = maximum severity of the occurrence of this diagnosis) [13]. This information was then forwarded to the study office. The type of homeopathic treatment, the use of any conventional therapy, as well as any referrals to other physicians were recorded on a continuous basis.

### Statistics

We calculated the severity of complaints (patients' assessments) and diagnoses (physicians' assessments), by averaging those four complaints/diagnoses named first for each patient during the baseline examination. At each follow-up (i.e. at 3, 12, and 24 months) the respective severity ratings were ascertained.

All results reported here are based on the intention-to-treat approach, i.e. each patient included in the study entered the final analyses. If patients dropped out or withdrew from the study we replaced the respective missing values: baseline complaints that had been cured were given a severity rating of 0 in all following examinations. For patients who died during the study, we inserted the maximum severity rating of 10. Consequently, any improvements are possibly underestimated, but certainly not overestimated. Other missing values were multiply imputed following the suggestions of Rubin [14]. Instead of filling in a *single *value as a substitute for a missing value, multiple imputation is a strategy by which each missing value is replaced simultaneously by a *set *of plausible values that represents the uncertainty about the right value to impute. Thus, each missing values is filled in several times generating several distinct data tables, each with a complete set of data relating to all patients without any missing values. These complete data tables are analysed separately using appropriate statistical models. Afterwards, the results from all statistical analyses are pooled to generate treatment effects and p-values. In our study we used the MCMC (Marcov chain Monte Carlo) replacement method and created 5 multiple imputed data tables.

For each imputed data set, treatment effects were estimated on the basis of generalised linear regression models. Generalised linear regression models are flexible and powerful tools to describe data from cohort studies [15]. They are generalisations of the well known and often applied multiple regression models which often appear to be too simple to describe longitudinal data adequately. A generalised linear model is best described by two components. First, the mean course of the outcome, and second, the correlation structure for measurements taken on the same individual at different times. In our study we divided the 2-year follow-up period into two parts. During the first part (0-3 month) we assumed that mean outcome increased (or decreased) linearly. For the second part (3-24 months) we assumed that the mean outcome increased (or decreased) according to a quadratic term. Moreover, we assumed that the correlation between two measurements could be described by a simple exponential function. This essentially means that the correlation only depended on the time span between the two measurements, and it decreased the larger this time span was. This approach is completely analogous to the recommendations given by Diggle, Liang, and Zeeger in their standard text book on the analysis of longitudinal data [15].

Usually, patients for clinical studies are not selected randomly from a target population but according to some selection criteria by which patients are sampled according to extreme measurements (high blood pressure, severe pain, low quality of life, ...). This inevitably leads to regression-to-the-mean, a statistical phenomenon that makes natural variation look like real changes [16]. Separating regression-to-the-mean effects from true treatment effects can be difficult but is at least feasible when the mean and the standard deviation of the target population are known [17]. In our study we made a rather conservative assumption on the target population (chronically ill patients seeking homeopathic care): to have the same quality of life as the general German population (i.e. a mean SF-36 score of 50 and a standard deviation of 10). From this we calculated the expected regression-to-the mean effect and compared it to the total change in quality of life, hereby applying Mee and Chua's modified t-test [17].

Although this study is explanatory by nature confidence intervals and p-values for change scores might be misinterpreted as statistical proof of hypotheses. We thus adjusted the results for all 12 outcomes (4 outcome measures times 4 time points) by applying Holm's procedure [18].

Standardized mean changes (effect sizes) were calculated by mean changes divided by standard deviation at baseline.

## Results

Patients were recruited between September 1997 and December 1999. Thirty-eight primary care physicians (33% of all participating doctors) reported treatment of adults over 70 years of age.

We have included 83 adults in this study, 59% (n = 49) women, age 70 - 87 years (mean 74.3 ± 3.1 SD) - and 41% men (n = 34), age 70 - 84 years (mean 73.2 ± 3.1) in the present analysis. For baseline characteristics of the study population see Table 1.

**Table 1:** Baseline characteristics of study sample of older adults (> 70 years) under homeopathic treatment (n = 83)

Gender (% female)	59.0
Age (years, mean ± SD)	73.9 ± 3.6

Living alone in household (%)	31.0

Living together with a partner (%)	66.3

Education (% attending school > 10 years)	28.9

Strong belief in homeopathy (%)	65.1

Duration of disease (years, mean ± SD)	11.5 ± 11.5
Intake of conventional drugs (%)	81.9

98.6 percent of all diagnoses were chronic with an average duration of 11.5 ± 11.5 years.

At baseline on average 3.0 ± 1.1 initial diagnoses (2.9 ± 1.1 of them chronic) were made. Fifty-seven percent of the patients had five or more complaints at baseline. The most frequent diagnoses were hypertension (21%) and sleep disturbances (16%). The most common diagnosis in women was sleep disturbance (22.4%) and in men hypertension (26.5%). For the most frequent diagnoses, their severity and duration is shown in Table 2.

**Table 2:** Most frequent diagnoses, disease severity and disease-duration in the study population of older adults (> 70 years) under homeopathic treatment

Disease (ICD 9)	Frequency % (n)	Disease severity (Physicians Assessment) (NRS 0-10) Mean ± SD	Disease Duration (years) Mean ± SD
Hypertension (401.9)	21 (17)	5.7 ± 1.7	11.1 ± 7.5

Sleep Disturbance (780.5)	16 (13)	7.1 ± 2.3	22.1 ± 25.8

Diabetes mellitus (250.0)	7 (6)	5.8 ± 1.5	8.0 ± 7.2

Sciatica (724.3)	7 (6)	5.7 ± 2.7	15.1 ± 16.3

Low Back Pain (724.2)	6 (5)	4.8 ± 2.3	19.0 ± 8.3

Osteoarthritis (715.3)	6 (5)	3.6 ± 1.5	9.5 ± 9.7

Depression (311.0)	6 (5)	5.8 ± 1.5	6.4 ± 6.7

All patients underwent an initial homeopathic consultation, lasting an average of 110 ± 36 minutes. During the 24-month observation period following the initial interview, patients consulted their physicians an average of 6.3 ± 6.3 times. The average period of treatment lasted 10 ± 9.1 months. Nine patients died during follow-up, the mean survival time was 376 days (range 6 to 725 days). Twenty patients stopped the homeopathic treatment. The reasons given were aggravation (n = 13), amelioration (n = 3), outcome unrelated reasons (n = 2) and no report (n = 2). During the study period eight patients (9.6%) were referred to non-study physicians (excluding dentists).

**Figure 1:**
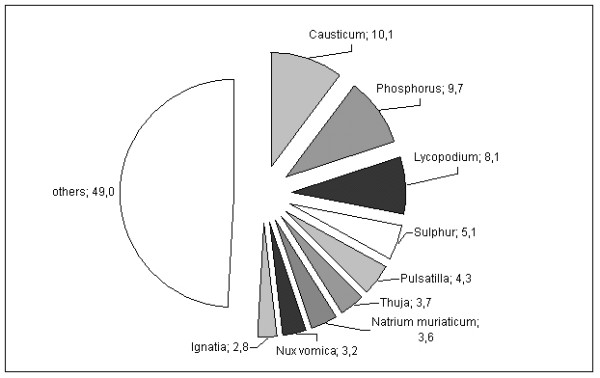
**Most frequently prescribed homeopathic drugs in the study sample of older adults (> 70 years; in %)**.

Each patient received on average 6.1 ± 5.3 (not necessarily different) homeopathic remedies. Prescriptions were given consecutively following the principles of classical homeopathy. More than half of all prescriptions were covered by 9 homeopathic remedies (Figure 1). The most frequently prescribed homeopathic potencies were C200 (31.3%), C1000 (13.5%), C30 (12.7%) and Q1 (9.9%). Eighty-two percent of the patients were taking medication at baseline (38% cardiovascular, 16% for central nervous system, 16% gastrointestinal and metabolic, 30% others), the number of prescribed drugs remained stable across 24 months (baseline: mean 2.6 ± 2.2 (SD); 3 months: 2.3 ± 2.0; 12 months: 2.0 ± 2.2; 24 months 2.3 ± 2.1).

**Figure 2:**
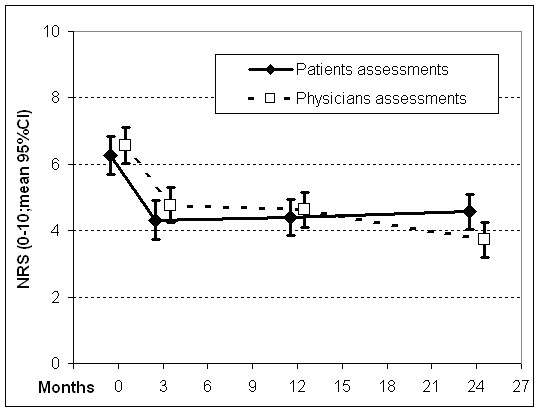
**Disease severity in study population of older adults (> 70 years) under homeopathic treatment over 24 months**.

The strongest clinical improvements of complaints were described by patients in the first three months, after which no further improvement was observed (Table 3, Figure 2). Complaint severity decreased significantly (p < 0.001) from 6.3 (95%CI: 5.7-6.8) to 4.6 (4.0-5.1) at 24 months. The physicians' assessments of diagnoses showed a more optimistic course for the long term, decreasing from 6.6 (6.0-7.1) to 3.7 (3.2-4.3; p < 0.001) (Table 3, Figure 2). Small but significant improvements in quality of life were observed on the SF-36 mental component scale during the first three months of treatment (p = 0.036). This significance however vanished if regression-to-the-mean was accounted for (p = 0.062, Mee-Chua-test). Overall the quality of life remained stable within the 24 months observation period. (Table 3). Again, no treatment effects could be confirmed that exceeded regression-to-the-mean effects (all p > 0.1).

**Table 3:** Outcome: Complaints, effect size, quality of life (estimated means and confidence intervals from the statistical model) in the study sample of older adults (> 70 years) under homeopathic treatment

STATUS	Month 0	Month 3	Month 12	Month 24		
SEVERITY (NRS, All Diagnoses, Mean and 95% CI)						
Complaints^a^	6.27 (5.70; 6.84)	4.31 (3.71; 4.90)	4.41 (3.87; 4.95)	4.57 (4.03; 5.10)		
Diagnoses^b^	6.57 (6.03; 7.11)	4.77 (4.23; 5.30)	4.63 (4.09; 5.16)	3.72 (3.19; 4.26)		

QUALITY OF LIFE (SF-36 Component Scores, Mean and 95% CI)						
Physical	38.37 (34.26; 42.47)	39.22 (35.11; 43.32)	38.86 (34.81; 42.90)	36.32 (32.29-40.35)		
Mental	46.22 (41.56; 50.88)	49.59 (44.91; 54.27)	47.62 (43.04; 52.21)	46.02 (41.46; 50.58)		

**CHANGE^c^**						
	**Months 0-3**	**p^c^**	**Months 0-12**	**p^c^**	**Months 0-24**	**p^c^**

SEVERITY (NRS, All Diagnoses, Mean and 95% CI)						
Complaints^a^	-1.96 (-2.67;-1.25)	< 0.001	-1.87 (-2.86; -0.88)	< 0.001	-1.71 (-2.83; -0.59)	< 0.001
Diagnoses^b^	-1.80 (-2.39; -1.21)	< 0.001	-1.94 (-2.71; -1.17)	< 0.001	-2.84 (-3.72; -1,96)	< 0.001

QUALITY OF LIFE (SF-36 Component Scores, Mean and 95% CI)						
Physical	0.85 (-1.20; 2.90)	1.00	0.49 (-3.08; 4.06)	1.00	-2.05 (-6.86; 1.76)	1.00
Mental	3.37 (0,62; 6.12)	0.036	1.40 (-3.29; 6.09)	1.00	-0,20 (-6.41; 6.01)	1.00

**STANDARDIZED MEAN CHANGE^c^**						
	**Months 0-3**	**p^c^**	**Months 0-12**	**p^c^**	**Months 0-24**	**p^c^**

SEVERITY (NRS, All Diagnoses, Mean and 95% CI)						
Complaints^a^	-1.21 (-1.65; -0.77)	< 0.001	-1.15 (-1.76; -0.54)	< 0.001	-1.05 (-1.74; -0.36)	< 0.001
Diagnoses^b^	-1.08 (-1.44; -0.72)	< 0.001	-1.17 (-1.64; -0.70)	< 0.001	-1.72 (-2.25; -1.19)	< 0.001

QUALITY OF LIFE (SF-36 Component Scores, Mean and 95% CI)						
Physical	0.05 (-0.07; 0.17)	1.00	0.03 (-0.17; 0.23)	1.00	-0.11 (-0.38; 0.16)	1.00
Mental	0.16 (0.02-0.30)	0.036	0.07 (-0.16; 0.30)	1.00	-0.01 (-0.32; 0.30)	1.00

^a ^Complaints = Patients' Assessment;^b ^Diagnoses = Physicians' Assessment

^c ^p-values and confidence intervals were multiply adjusted to maintain an overall level of significance of 5%. However, due to the nature of this study they should only be interpreted as exploratory.

NRS = Numerical Rating Scale: 10 = maximum, 0 = no complaints. Negative change in NRS indicates improvement. Positive quality of life change indicates improvement.

Absolute effect size > 0.8, large; > 0.5, medium; > 0.2, small.

## Discussion

In a prospective multicentre observational study with qualified homeopathic physicians in daily practice, we documented the homeopathic and conventional treatment with its outcome in 83 elderly patients over 24 months.

The study provided information on the course of disease in elderly patients receiving homeopathic treatment, as assessed by both patients and physicians.

Patient and physician assessments of the severity of the complaints consistently demonstrated substantial improvements following homeopathic treatment, which were maintained through 24 months of follow up. Overall the quality of life and the number of medicines taken remained stable within the 24 months observation period.

To our knowledge, the present study is the first to systematically evaluate the range of diagnoses and therapies in classical homeopathic medical practices in Germany and Switzerland in patients over 70 years old. A strength of this study is that patients with all diagnoses were included. Therefore, no disease-specific measurement instruments could be used. To assess the severity of different medical complaints, there is no other generally accepted measuring instrument available. Instead numerical rating scales [12] were applied, which would allow for the determination of the severity of the complaint in a diagnosis-independent manner. However, our data may be helpful in the planning of further research on homeopathy including randomized clinical trials on the effectiveness of individually chosen homeopathic remedies. These trials however should include tailored instruments which measure treatment effects more specifically than the rather global measures we employed in this study.

Our study was not designed to assess the effectiveness of the homeopathic remedies, therefore the chosen methodology did not include a control group, randomization or blinding and patients could use additional conventional therapies. Thus, the observed improvement can be attributed to a wide range of possible reasons as it is known for complex interventions. Our findings might indicate that homeopathic medical therapy may play a beneficial role in the long-term care of older adults with chronic diseases and studies on comparative effectiveness are needed to evaluate this hypothesis.

There might be some selection bias because the homeopaths belong to a group using only classical homeopathy. Other forms of homeopathy, for example, clinical homeopathy are more focused on the primary disease symptoms, treat more often acute diseases and have shorter case taking and use a smaller range of homeopathic drugs. In addition information bias might be possible because we follow the assumption that missing values are per random, this might result in a underestimation or overestimation of effect. A further limitation of our study might be that 65.1% of our elderly patients had a strong belief in homoeopathy. This might have triggered the therapeutic outcome due to high therapeutic expectations.

It is of special note that in this study the average severity of the chronic diseases was reduced by approximately 30% after only three months of homeopathic treatment, and remained at about this level during the follow-up period. Physician assessments tended to be more positive than patient assessments. This is supported by the fact that patients did not visit the study physicians when they were feeling their worst, but rather after a long waiting period. The interpretation of our quality of life results is difficult. The SF-36 baseline values of our homeopathic patients were lower than in the German normal population of the same age group (physical component score - difference: 1.5 and mental component score: 6.2) [13]. In the German population SF-36 values for physical and mental component score decrease with increasing age [13]. Based on this the absence of a decrease in quality of life in our elderly patients might be interpreted in a positive way.

We were unable to confirm the common notion that homeopathy is frequently used for trivial complaints or diseases. The duration of disease in our study patients was very long and their symptoms were, on average, of moderate severity. The spectrum of complaints is clearly age-related and differs from the total sample of all adult patients: in women (n = 2017) the most frequent diagnoses were migraine (9.7%), headache (9.1%), sleep disturbances (7.5%) and eczema (7.3%); in men (n = 834) allergic rhinitis (10.3%), eczema (7.8%), hypertension (7.7%) and sleep disturbances (6.5%) [10].

If we compare our results for elderly patients with other homeopathic outcome studies [9-11,19-21] there is a common and clinically relevant improvement of the severity of complaints for most patients over all age-groups within the first few months of homeopathic treatment. For all 3981 patients in our cohort severity of disease decreased significantly from 6.2 (SD ± 1.7) to 3.0 (± 2.2), the initial improvement being stable during the 24 month follow-up. Major improvements in quality of life could only be observed in children, but not in adults. In our elderly subgroup we can confirm these results.

## Conclusion

The findings of our study demonstrate that elderly patients who seek homeopathic treatment are primarily those suffering from long-standing, chronic disease.

The severity of complaints decreased markedly in the first 3 months of treatment. However, the quality of life remained stable. Our findings might indicate that homeopathic medical therapy may play a beneficial role in the long-term care of older adults with chronic diseases and studies on comparative effectiveness are needed to evaluate this hypothesis.

## Competing interests

The authors declare that they have no competing interests.

## Authors' contributions

MT participated in the interpretation of the results and drafted the manuscript. RL participated in its design and performed the statistical analysis. KS participated in the interpretation of the results. SW conceived the study, participated in its design and statistical analysis and had the overall scientific responsibility. CW participated in the design of the study, coordination and statistical analysis. All authors helped to draft the manuscript, read and approved the final version.

## Pre-publication history

The pre-publication history for this paper can be accessed here:

http://www.biomedcentral.com/1471-2318/10/10/prepub
